# Altered glucose and lipid homeostasis in liver and adipose tissue pre-dispose inducible NOS knockout mice to insulin resistance

**DOI:** 10.1038/srep41009

**Published:** 2017-01-20

**Authors:** Babu Nageswararao Kanuri, Jitendra S. Kanshana, Sanjay C. Rebello, Priya Pathak, Anand P. Gupta, Jiaur R. Gayen, Kumaravelu Jagavelu, Madhu Dikshit

**Affiliations:** 1 Pharmacology Division, CSIR-Central Drug Research Institute, Lucknow - 226031, India; 2 Academy of Scientific and Innovative Research, New Delhi - 110001, India; 3 Pharmacokinetics and Metabolism Division, CSIR-Central Drug Research Institute, Lucknow - 226031, India

## Abstract

On the basis of diet induced obesity and KO mice models, nitric oxide is implied to play an important role in the initiation of dyslipidemia induced insulin resistance. However, outcomes using iNOS KO mice have so far remained inconclusive. The present study aimed to assess IR in iNOS KO mice after 5 weeks of LFD feeding by monitoring body composition, energy homeostasis, insulin sensitivity/signaling, nitrite content and gene expressions changes in the tissues. We found that body weight and fat content in KO mice were significantly higher while the respiratory exchange ratio (RER), volume of carbon dioxide (VCO_2_), and heat production were lower as compared to WT mice. Furthermore, altered systemic glucose tolerance, tissue insulin signaling, hepatic gluconeogenesis, augmented hepatic lipids, adiposity, as well as gene expression regulating lipid synthesis, catabolism and efflux were evident in iNOS KO mice. Significant reduction in eNOS and nNOS gene expression, hepatic and adipose tissue nitrite content, circulatory nitrite was also observed. Oxygen consumption rate of mitochondrial respiration has remained unaltered in KO mice as measured using extracellular flux analyzer. Our findings establish a link between the NO status with systemic and tissue specific IR in iNOS KO mice at 5 weeks.

Nitric oxide (NO), an important gaseous signaling molecule, regulates numerous physiological processes having its synthesis being regulated by Ca^+2^ dependent nitric oxide synthases (eNOS and nNOS) and/or Ca^+2^ independent inducible nitric oxide synthase (iNOS)^1^,^2^. Intracellular compartmentalization of iNOS is observed in metabolic organs such as primarily kupffer cells, periportal hepatocytes, cytoplasm and plasma membrane of white adipose tissue (WAT) adipocytes, cell membrane (sarcolemma) and cytoplasm (sarcoplasm) of skeletal muscle myocytes^3^,^4^. However, under normal conditions constitutive expression of iNOS is predominant in kupffer cells and minor in hepatocytes^4^. Studies reported so far suggest a prominent role of NO/NOS in the pathogenesis of metabolic syndrome, insulin resistance (IR), obesity and diabetes type I/II^5^,^6^,^7^. Augmented visceral fat deposition is an independent risk factor for IR^8^ and clinical studies have shown that higher body mass index in obese patients correlates positively with IR, hyperinsulinemia and glucose intolerance^8^,^9^.

*In vitro* studies on C2C12 myotubes using NO donor (GSNO) or ectopic iNOS expression demonstrated IRS-1 degradation^10^. High fat diet (HFD) fed obese (DIO) and ob/ob mice exhibited elevated iNOS expression in liver, skeletal muscle and adipose tissue^5^,^6^ and also the inactivation of insulin receptor β/insulin receptor substrate-1 (IRS-1) and AKT^11^,^12^. Treatment of ob/ob mice with iNOS selective inhibitor reversed fasting hyperglycemia^5^. Moreover, iNOS KO mice fed with 30% fructose for 8 weeks were reported to be protected against hepatic steatosis and inflammation^13^. However, selective over expression of iNOS in liver increased obesity/IR, hepatic IR, and mild hyperglycemia^7^. On the contrary, rats fed on chow diet and NOS inhibitors showed increased fat deposition, and enhanced level of circulating and hepatic lipids^14^,^15^,^16^. Interestingly, HFD fed iNOS KO mice were protected from diet induced systemic IR even though they exhibited elevated body weight, fat deposition and fasting hyperglycemia^17^. Hyper-insulinemic-euglycemic (HE) clamp study demonstrated a significantly reduced glucose uptake by the skeletal muscle in aged mice, but not in the young adult iNOS KO mice^18^. Studies published so far thus found iNOS, to be both protective and detrimental in IR and insulin signaling. It is evident that most of the studies conducted on iNOS KO mice did not examine the systemic IR and insulin signaling in all the metabolic organs but monitored insulin signaling/IR in only one or two organs after feeding standard low fat/chow diet or HFD (55–60%) with diverse regimens (16–18 weeks)^17^,^19^,^20^. However, parameters related to energy homeostasis such as food intake, energy utilization and physical activity, and genes responsible for glucose and lipid metabolism were not profiled in these studies^17^,^20^.

These contradictory outcomes associated with iNOS studies and IR could also be due to the use of variable protocols such as amount of glucose (1–2 g) and insulin (0.35 to 1.5 IU) administered for monitoring the tolerance, duration of fasting (4–12 h), glucose/insulin levels and the types of diets (10–60% kcal in energy)^17^,^20^. The present study was undertaken to address the anomalies in the role of iNOS in the incidence of IR by using WT and iNOS KO mice fed on low fat diet (LFD) (often used as isocaloric diet to assess high fat diet induced dyslipidemia) for 5 weeks. Circulating glucose, insulin and lipids were measured in the both on ad libitum fed and on fasted (6 h & 12 h time points) mice. To systematically assess incidence of IR in the iNOS KO mice, ITT, GTT, PTT, as well as insulin signaling proteins and expression of selective genes involved in the glucose and lipid metabolism were measured by Western blotting and RT-PCR respectively. Further, WT and iNOS KO mice were assessed for total circulatory nitrite, tissue specific nitrite (liver, adipose and skeletal muscle) and NOS (eNOS, nNOS) isoforms gene expressions followed by hepatic and adipose tissue isolated mitochondrial OCR and UCP2 gene expressions. Our results demonstrate the incidence of tissue specific (liver and adipose tissue but not skeletal muscle) and systemic IR as a resultant of reduced NO availability in iNOS KO mice fed with LFD for 5 weeks.

## Results

### Weight and body composition of WT and iNOS KO mice

**Figure 1:**
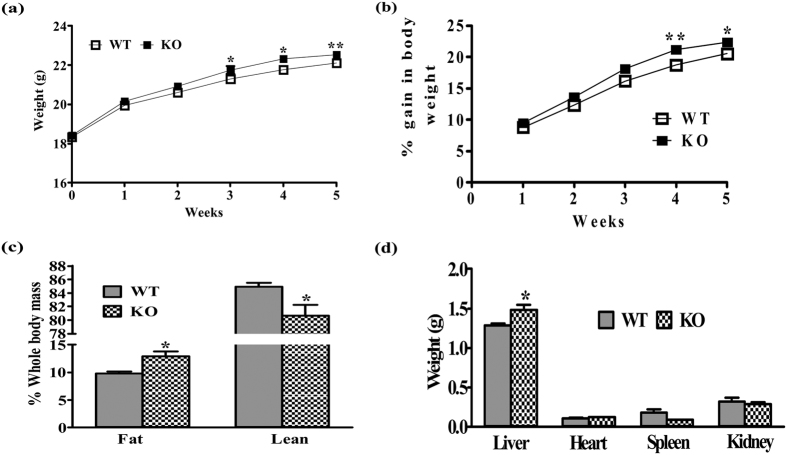
Gross parameters in wild type (WT) and iNOS knockout (KO) mice. **(a)** Body weight, **(b)** % increase in body weight, **(c)** % change in whole body fat/lean mass and **(d)** tissue weights (n = 6–9). Comparison between groups was done by Student’s t test. *p < 0.05 and **p < 0.01 vs WT.

Significant gain in body weight was noticed in iNOS KO mice from third week of LFD feeding and it continued until five weeks (Fig. 1a,b). Whole body fat was enhanced while lean mass was reduced as measured by ECHO-MRI (Fig. 1c). Liver weight was markedly increased, however no significant change in heart, spleen and kidney weight was observed in iNOS KO mice as compared to WT (Fig. 1d).

### Food intake, energy utilization and physical activity

**Figure 2:**
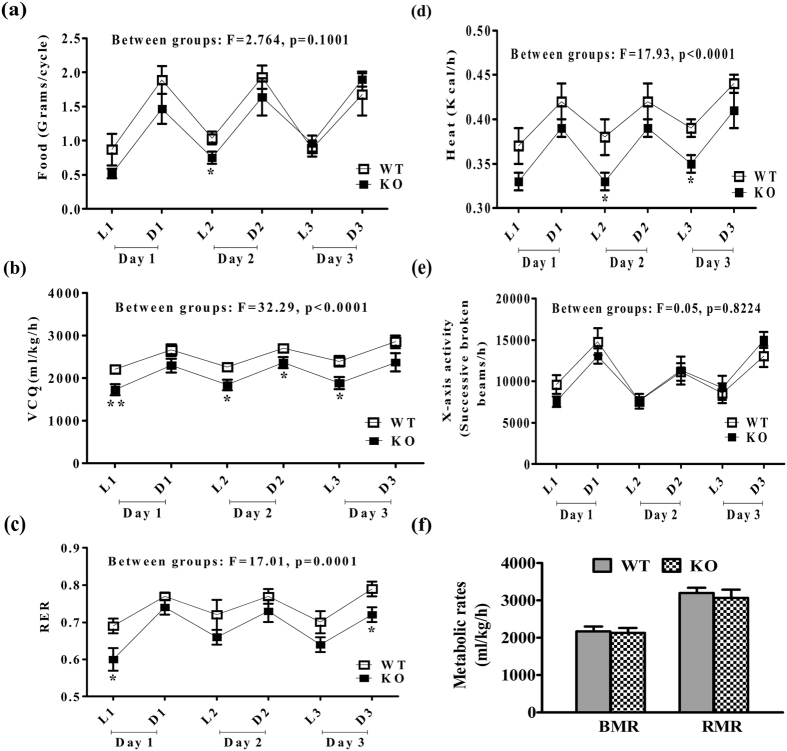
Food intake, energy utilization and physical activity in WT and iNOS KO mice. **(a)** Food consumption, **(b)** VCO_2_, **(c)** RER, **(d)** heat production, **(e)** X-axis movement and **(f)** metabolic rates (n = 6–8). L, D represents Light and Dark phases of a single day while 1, 2 and 3 indicates days of experiment. Comparison between groups was done by two way ANOVA followed by Bonferroni post hoc test (WT vs KO). *p < 0.05 and **p < 0.01 vs WT.

Food intake and physical activity in iNOS KO mice was not affected during 5 weeks of LFD compared to WT (Fig. 2a,e). However, VCO_2_, RER and heat production were reduced significantly as compared to WT controls (Fig. 2b–d). Furthermore, metabolic rates as indicated by BMR and RMR were almost similar in KO and WT mice (Fig. 2f).

### Glycemic control and insulin sensitivity

**Figure 3:**
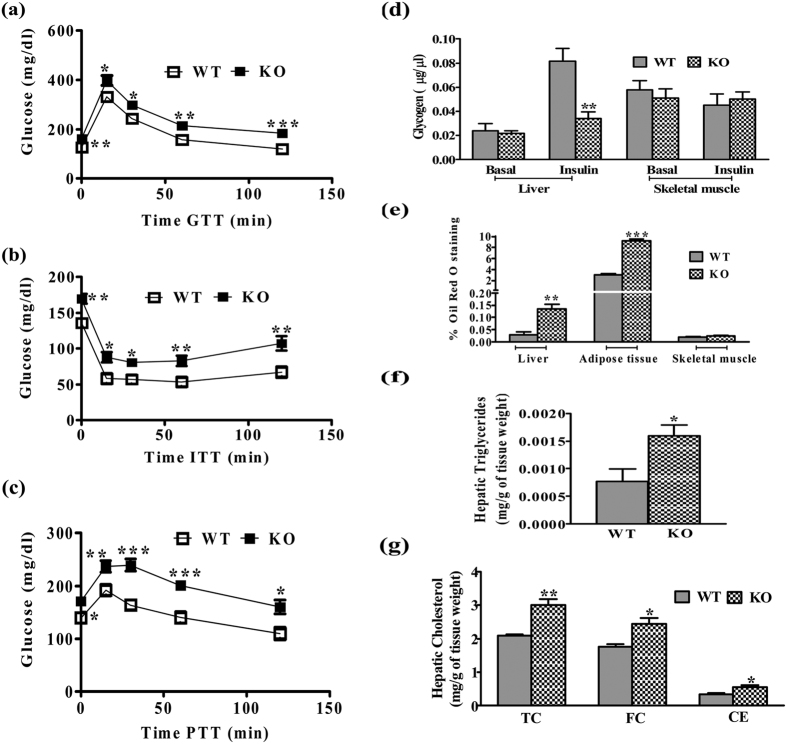
Systemic glycemic control and tissue biochemistry. **(a)** GTT (n = 8), **(b)** ITT (n = 10), **(c)** PTT (n = 9), **(d)** hepatic and skeletal muscle glycogen content (n = 5), **(e)** hepatic, adipose tissue and skeletal muscle Oil red O staining (n = 5), **(f**,**g)** hepatic triglycerides and cholesterol (n = 5). Comparison between groups was done by Student’s t test. *p < 0.05, **p < 0.01 and ***p < 0.001 vs WT.

**Figure 4:**
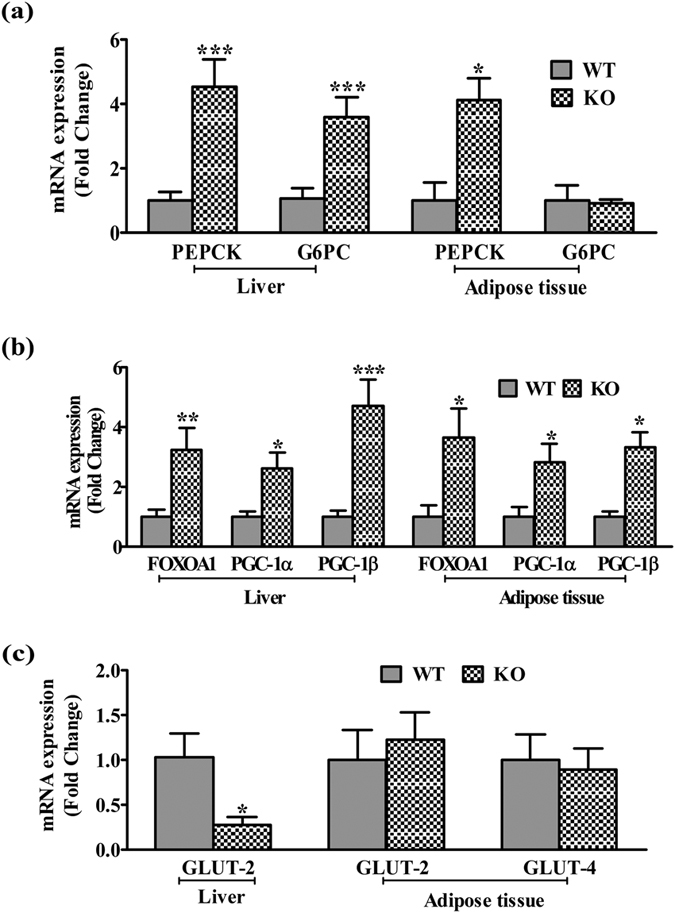
qPCR expressions of **(a)** Enzymes and **(b)** Transcriptional regulators involved in hepatic (n = 9–11) and adipose tissue (n = 4) gluconeogenesis, and **(c)** Glucose transporters (GLUTs, n = 4). Comparison between groups was done by Mann-Whitney U test. *p < 0.05, **p < 0.01 and ***p < 0.001 vs WT.

A significant increase in serum insulin and glucose levels in KO mice indicated a compensatory insulin action to maintain euglycemia for 6 h fasting (Table 1). Subsequent studies in overnight fasted group showed no significant change in glucose and insulin levels. Marked difference was observed in HOMA-IR and QUICKI but not HOMA-B between WT and KO mice, indicated systemic IR with functional β-cells in iNOS KO mice^21^,^22^ (Table 1). Subsequently, tissue insulin sensitivity and glucose disposal to metabolic organs were measured using glucose and insulin tolerance tests. A significant increase in the blood glucose level in iNOS KO mice was found untill 2 h after intra-peritoneal (i.p.) glucose or pyruvate administration (GTT & ITT) (Fig. 3a,b). Similarly, PTT analysis for insulin sensitivity was also adversely affected in WT and KO mice, suggesting a persistent hepatic gluconeogenesis (Fig. 3c). Real time PCR data indicated that hepatic and adipose tissue gluconeogenetic enzymes (PEPCK, G6PC) and their transcriptional regulators (FOXOA1, PGC-1α and PGC-1β) were found to be augmented by 2–3 folds (Fig. 4a,b). Insulin stimulated hepatic glycogen level (Fig. 3d) was significantly reduced, but skeletal muscle glycogen content was not altered (Fig. 3d). qPCR analysis of glucose transporters in WT and KO mice showed a significant decrease in hepatic GLUT-2 with unaltered adipose tissue GLUT-2, 4 (Fig. 4c).

### Insulin signaling proteins

**Figure 5:**
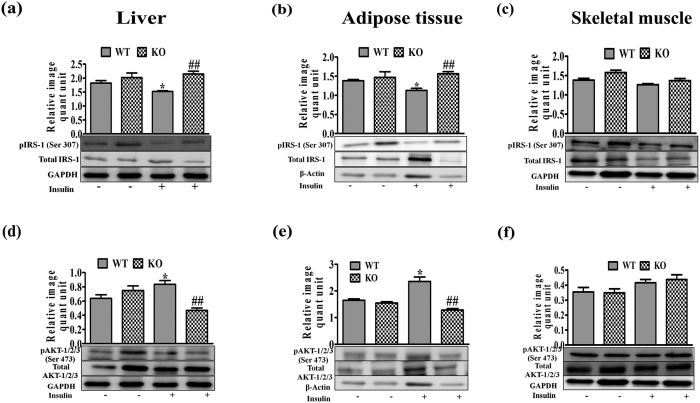
Effect of 5 weeks LFD feeding on insulin signaling proteins in liver, adipose and skeletal muscle tissues. Western blots analyses of **(a–c)** pIRS-1 (Ser 307) and **(d–f)** pAKT-1/2/3 (Ser 473) in liver, adipose and skeletal muscle tissues. Bar diagrams represent mean ± SEM of the change in image density (n = 3). Comparison between groups was done by Student’s t test. *p < 0.05 vs WT Basal and ^##^p < 0.01 vs WT Insulin.

To gain further insight, phosphorylation status of IRS-1 (Ser 307) and AKT-1/2/3 (Ser 473) was monitored during insulin stimulated conditions in liver, skeletal muscle and adipose tissue^23^. IRS-1 phosphorylation on Ser 307 was enhanced in liver and adipose tissues but not in skeletal muscle (Fig. 5a–c). AKT-1/2/3 phosphorylation at Ser 473 being prerequisite for normal insulin signaling was reducedin liver and adipose tissues, but not in skeletal muscle of KO mice (Fig. 5d–f).

### Systemic cytokine status

**Figure 6:**
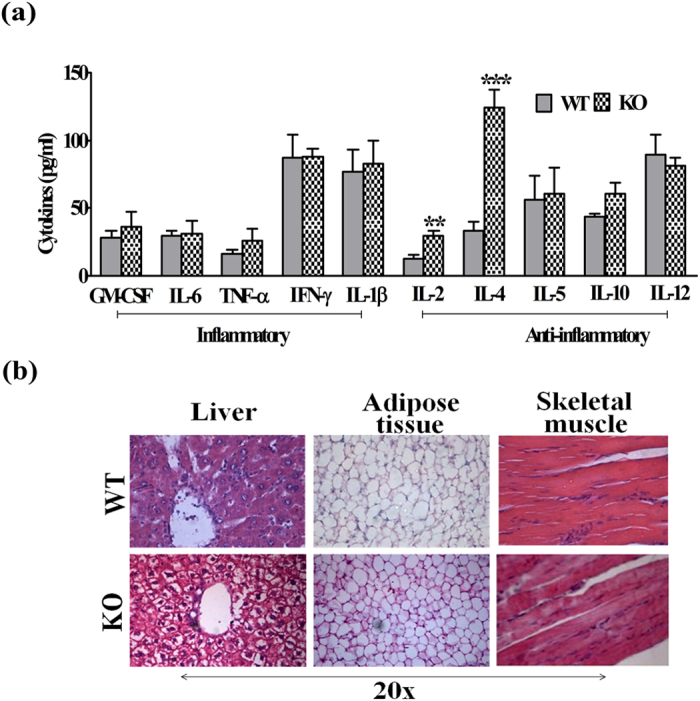
(**a**) Serum cytokine status (n = 6–8) and **(b)** HE staining in WT and iNOS KO mice. Comparison between groups was done by Student’s t test. **p < 0.01 and ***p < 0.001 vs WT.

Previous studies have shown that sub-acute inflammation is crucial for metabolic disorders. We thus measured the circulating inflammatory and anti-inflammatory cytokines in WT and iNOS KO mice. Serum cytokine profile analyses indicated only a trend of increase in IL-1β, which was statistically not significant (p > 0.05). However, the levels of other pro-inflammatory cytokines (IFN-γ, GM-CSF, IL-6, TNF-α) were remain unchanged. On the contrary, anti-inflammatory cytokines, IL-2 and IL-4 were significantly increased in iNOS KO mice (Fig. 6a).

### Systemic and hepatic lipid content

**Figure 7:**
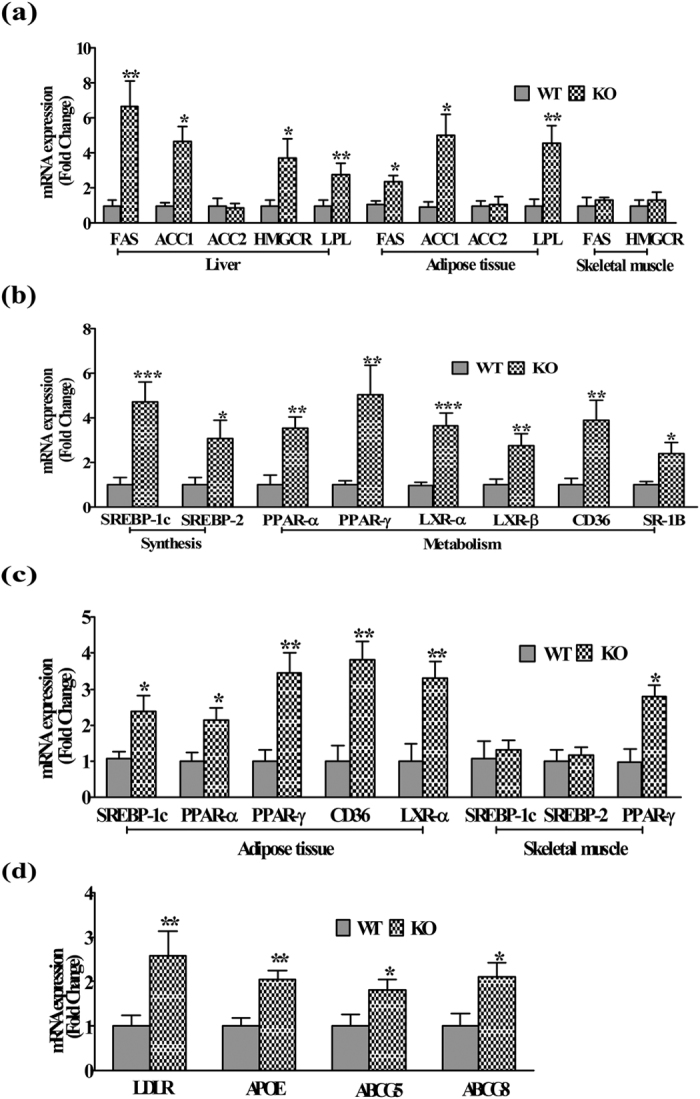
Hepatic and adipose tissue lipid modulating genes. **(a)** qPCR expressions of enzymes and **(b**,**c)** key regulators of hepatic (n = 8–12), adipose tissue (n = 6–10), skeletal muscle (n = 4) lipid metabolism, and **(d)** hepatic lipid excretion (n = 10). Comparison between groups was done by Mann-Whitney U test. *p < 0.05, **p < 0.01 and ***p < 0.001 vs WT.

iNOS KO mice fed ad libitum on LFD exhibited a profound increase in the serum TG, TC, and NEFA during fasted as well as in fed conditions (Table 1). The increase in serum lipids was more pronounced in overnight fasted iNOS KO mice as compared to 6 h fasted or ad libitum fed mice. However, no significant change was observed in the serum LDL and HDL in fasted or ad libitum fed mice. Pathophysiology of IR is often associated with excessive lipid accumulation in adipose tissue, lipolysis and hepatic de novo lipogenesis^24^. Morphological assessment by HE staining detected the presence of white spots around bluish nucleus on pink cytoplasmic background in iNOS KO mice liver (Fig. 6b). Oil red O staining of three metabolic organs indicated a significant lipid accumulation in liver and adipose tissue but not in skeletal muscle of iNOS KO mice (Fig. 3e). Estimation of hepatic triglyceride and total cholesterol levels confirmed increased lipid accumulation in iNOS KO mice (Fig. 3f,g). Further, the qPCR analysis of genes involved in hepatic lipogenesis (FAS, ACC1 & 2, HMGCR, SREBP-1C and SREBP-2) and metabolism (PPAR-α, γ, LXR-α, β, LPL, ApoE, LDLR, ABCG5 and ABCG8) indicated 2–4 fold augmentation in the expression of genes coding for triglyceride (FAS, ACC1 & 2, SREBP-1C), total cholesterol (HMGCR & SREBP-2) synthesis and β-oxidation (PPAR-α, γ, LXR-α, β) suggesting that iNOS KO mice are prone to hepatic steatosis (Fig. 7a,b). Surprisingly, augmentation in lipid efflux genes (ApoE, LDLR, ABCG5 & ABCG8) in iNOS KO mice liver suggests simultaneous increase in the lipid efflux (Fig. 7d). Subsequently, qPCR analysis of adipose tissue and skeletal muscle lipid metabolism associated genes revealed a significant increase in genes coding for lipogenesis and lipolysis in former but not in latter tissue (Fig. 7c).

### NO levels in plasma, liver, adipose and skeletal tissue

**Figure 8:**
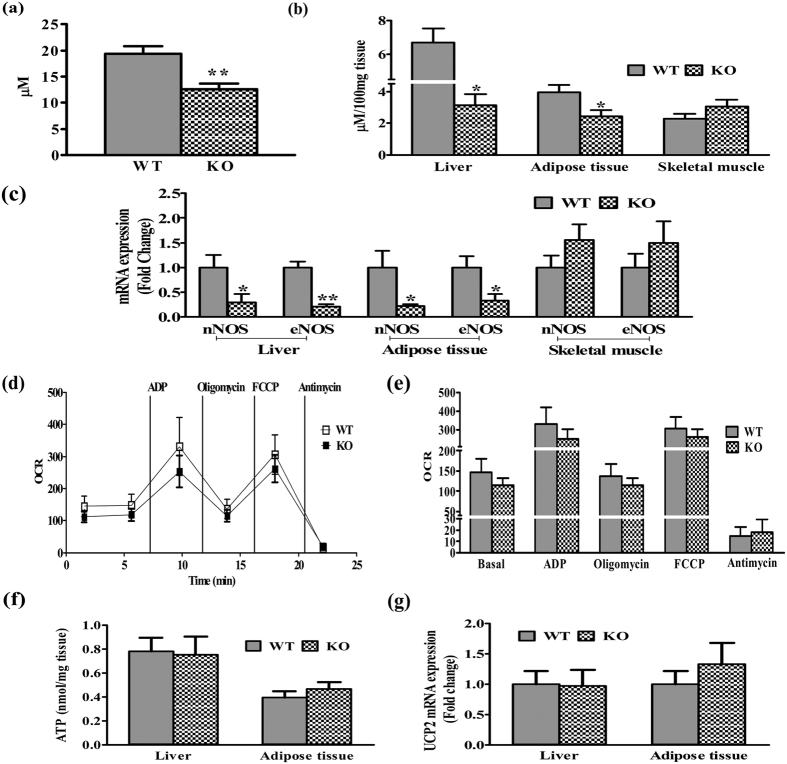
NO dependent systemic IR in iNOS KO mice. Total nitrite levels in **(a)** Plasma and **(b)** metabolic tissues (liver, adipose tissue and skeletal muscle) (n = 5), qPCR expressions of **(c)** eNOS, nNOS and **(g)** UCP2 (n = 6), **(d**,**e)** hepatic mitochondrial OCR (n = 3), and **(f)** tissue ATP (n = 5) in 5 weeks LFD fed WT and iNOS KO mice. Comparison between groups was done by Student’s t test except qPCR studies where Mann-Whitney U test was applied. *p < 0.05 an **p < 0.01 vs WT.

We checked circulatory and tissue NO content in insulin resistant iNOSKO mice after 5 weeks of LFD feeding. Nitrite content in plasma (Fig. 8a), liver, and adipose (Fig. 8b) were significantly reduced except in the skeletal muscle (Fig. 8b). Further, iNOS deletion led to attenuated eNOS and nNOS gene expressions in liver and adipose tissues but not in skeletal muscle. These results signify that inhibition of iNOS is the major regulator of NO, eNOS and nNOS in tissue insulin resistance (IR) resulting in systemic IR (Fig. 8c). Previous reports suggest that alterations in tissue NO affects mitochondrial function and biogenesis^3^. Mitochondrial basal oxygen consumption rate (OCR) in isolated hepatic (Fig. 8d,e) and adipose tissue (Fig. S1a,b) showed a decreased trend. However, a significant difference was observed in the adipose tissue of KO mice. Nonetheless, the OCR and ECAR (data not shown) were not affected in both the liver and adipose tissue. Further, 5 weeks LFD fed WT and KO mice demonstrated similar hepatic and adipose tissue ATP levels (Fig. 8f) and UCP2 (Fig. 8g) gene expression.

## Discussion

Obesity linked IR is a multi-factorial pathology. Enhanced oxidative (ROS)/nitrosative (RNS) stress and inflammatory cytokinesis is well documented during the initial stages of IR^25^,^26^,^27^. The present study was undertaken to extensively evaluate incidences of IR by monitoring weight & fat gain, food intake, physical activity, energy utilization, circulating lipids, systemic insulin resistance, insulin signaling in the metabolic organs and the level of circulating inflammatory cytokines in LFD fed iNOS KO mice. Low fat diet as an isocaloric diet is often used in studies conducted on HF induced dyslipidemia, and was also used for mice feeding in this study^28^. Moreover, an optimum duration of five weeks feeding was considered appropriate in the present study, as alterations in redox status are expected to be evident during initiation of IR. Weight gain in iNOS KO mice correlated with body fat and lean mass as measured by ECHO-MRI^29^. Perreault and Marette, like us observed significant body weight gain after 16 weeks of 55% HFD feeding to iNOS KO mice^17^. Epididymal and retroperitoneal white adipose tissue (WAT) in iNOS KO mice were significantly increased in 18 weeks fed 55% HFD diet^17^,^19^. Surprisingly, Becerril *et al*. had observed a significant reduction in body weight and total WAT after 12 weeks of chow diet feeding in iNOS KO mice^30^. On the contrary, other studies reported similar level of weight gain in WT and iNOS KO mice fed on chow diet up to 22 months or HFD (55–60% kcal) for 18 weeks^18^,^19^,^20^. Our study is strengthened by monitoring other tissue weights apart from enhanced liver weight in line with other published data^17^,^19^. In the present study, significant reduction in RER, VCO_2_, and heat production was noticed with no change in food intake and physical activity in iNOS KO mice, indicating altered energy utilization. A single study on iNOS KO mice measured similar parameters, but failed to find alterations in VO_2_ and RER following HF feeding for 18 weeks^19^. Nevertheless, parameters measured by us following LFD feeding in WT mice were comparable to the reported values^6^,^19^,^28^,^30^,^31^,^32^. HF feeding to WT mice exhibited augmented serum lipids, altered insulin signaling, and expression of metabolically important enzymes/transcription factors^31^. iNOS KO mice exhibited altered expression of metabolically important genes (Figs 4 and 7), implying a regulatory role of iNOS/NO in glucose and lipid homeostasis, as reported for eNOS and nNOS KO mice^33^,^34^,^35^,^36^,^37^,^38^,^39^.

Treatment of rodents with non-specific pharmacological NOS inhibitors like L-NNA or L-NAME for 2–8 weeks resulted in enhanced body fat, serum lipids and IR as observed by us in iNOS KO mice^14^,^15^,^16^. Genetic knockout of single (eNOS/nNOS)^33^ or triple (eNOS, nNOS and iNOS)^40^ null mice are pre-disposed to IR; on the contrary, iNOSKO have so far remain inconsistent^17^,^18^,^19^,^20^. Diet induced obesity and IR studies in iNOS KO were conducted mostly after 16–18 weeks of HF diet feeding^17^,^19^,^20^. However, in the present study iNOS KO mice exhibited a significant change in lipids, glucose and insulin even after 5 weeks of LFD feeding (Fig. 3 and Table 1). Long term (16–18 weeks) feeding with 55% HFD led to fasting hyperglycemia (5 h) with low insulin (Perreault and Marette) which was inconsistent with Dallaire *et al*., though similar dietary regimens were used^17^,^19^. In the present study we measured most of the parameters (lipids, insulin, glucose and insulin signaling proteins), by using three different feeding time points (6 h, 12 h fasting and food ad libitum). Significant changes in glucose and insulin were evident only after 6 h fasting and in fed condition but not after overnight fasting, while serum lipids remain altered. Normal blood glucose and serum insulin in overnight but not in 6 h fasted KO mice signify their pre-diabetic insulin resistant state. This might be attributed to some secondary systemic effects rather than primary iNOS absence which needs to be studied^7^. None of the studies on iNOS KO mice found systemic IR and β-cell functionality, as observed by us using HOMA indices. Gene profile of glucose and lipid metabolism as seen in this study was also not examined by others. We found both hepatic gluconeogenesis and altered glycogen content in iNOS KO mice as compared to their WT counterparts. Further, data from tolerance tests (GTT, PTT and ITT), skeletal muscle glycogen content and Western blots of insulin signaling confirmed that mice lacking iNOS had altered body glucose homeostasis and insulin sensitivity in hepatic and adipose tissue but not in skeletal muscle. Most of the previous studies on iNOS KO mice performed GTT and ITT and never assessed for PTT^17^,^19^,^20^. All the reported studies involving whole body iNOS deletion but not myeloid specific KO, failed to observe systemic IR in iNOS KO mice^17^,^19^,^20^. The difference in the present study and other reports is of the duration of feeding (16–18 weeks), composition of the diet (55 to 60% kcal) and the protocols (Dose and fasting time for biochemical estimations) used to assess IR^17^,^19^,^20^. There are two studies in the literature, which assessed IR signaling proteins [p-IRS (AKT 473) and/or PI3K activity] at 16 or 18 weeks of 55% HFD feeding^17^,^19^. Our results highlights the presence of systemic IR which the other studies neglected, nonetheless our results concur with published reports on hepatic and adipose tissue IR and the absence of skeletal muscle IR in iNOS KO mice^17^,^19^. A significant decrease in plasma total nitrite in insulin resistant iNOS KO mice, which is in line with the earlier published report^18^. Perreault and Marette, observed a marked decrease in eNOS gene expression in chow diet fed iNOS KO mice WAT but not in skeletal muscle^17^. On the contrary, nNOS expression was reduced in muscle and was not expressed in WAT^17^. Variations in the expression of the respective genes might be related to the sensitivity of the technique used for gene assessments. The present study highlights the requirement of optimal iNOS levels in the maintenance of normal glucose/lipid metabolism and insulin sensitivity.

Hyperinsulinemic euglycemic (HE) clamp study in the adult and old mice showed a significant decrease in the whole body and skeletal muscle glucose uptake in old (22 months) but not in adult (7 months) iNOS KO mice^18^. Conversely, improved muscle glucose uptake in the isolated soleus muscles of young and old iNOS KO mice has also been published recently^41^. These studies highlight that skeletal muscle cell glucose utilization and metabolism is differently regulated in iNOS KO mice. Though we did not carry out glucose uptake studies, it was however evident that the change in skeletal muscle glycogen, IR marker proteins and qPCR gene expressions was statistically not significant. Glutathione and NO are imperative for insulin sensitivity, its modulation in iNOS KO mice is however an unexplored area so far and needs attention^42^. Reactive oxygen species (ROS) and H_2_O_2_ also act as a secondary messengers in insulin signaling and mimic insulin effects^43^, their excessive production has been implicated in IR pathophysiology^43^. The significance of ROS and associated redox modulators in insulin resistant iNOS KO mice remains to be explored. Obesity linked IR is described as a sub-acute inflammatory disorder and inflammatory cytokines are the crucial players^44^. Nevertheless, protective role of iNOS against tissue degradation has recently been reported^45^. iNOS KO macrophages exhibited significantly higher basal generation of H_2_O_2_ as well as LPS induced TNF-α and MIP-2 production, suggesting dual role of iNOS in inflammation^46^. Status of inflammatory cytokines in the current study, dissociates inflammation with IR in iNOS KO mice as we failed to observe any significant change in these cytokines. This is also in agreement with a previous study where standard chow fed adult iNOS KO mice showed no significant variation in muscular and adipose pro-inflammatory cytokine gene expressions^18^. In old age mice (22 months), iNOS KO mice showed significant increase in pro-inflammatory and a decrease in anti-inflammatory cytokine gene expressions^18^. The altered cytokine levels in old but not in adult iNOS KO mice coincided with reduced glucose uptake, suggesting that mice lacking NOS2 were pre-disposed to IR with the advancement of age^18^.

As evident from hepatic insulin signaling data, we and others^17^,^19^ have not found protection in the hepatic tissue against IR in the insulin signaling. We extended this observation by measuring hepatic lipids which was profoundly increased in KO mice. Serum lipid levels in KO mice were also enhanced with fasting, possibly due to high rates of lipid oxidation and systemic IR^47^. Moreover, IR at liver and adipose tissue level was substantiated by measuring genes responsible for lipid metabolism (synthesis, breakdown and/or efflux) in iNOS KO mice. Further, data obtained from Oil red O staining and lipogenic gene expressions rule out the role of skeletal muscle in pre-disposing iNOS KO mice to systemic IR. In the present study, we report for the first time that functional markers of liver or kidney injury (ALT, T. Bilirubin and Creatinine) were not altered at 5 weeks of LFD between both WT and iNOSKO mice. It is clearly evident that LFD fed iNOS KO mice exhibited impairment in glucose and lipid metabolism correlating well with altered body composition and energy homeostasis. Excessive iNOS was often linked with IR^5^,^6^,^7^, on the contrary, even its absence has been associated with systemic IR.

It is well documented that NO at physiological concentrations acts as an endogenous messenger in maintaining systemic glucose and lipid homeostasis by promoting their utilization but not synthesis^3^. Gene manipulation of endothelial NOS (eNOS) in mice has highlighted its importance in maintaining systemic glucose and lipids by regulating metabolism in three major metabolic organs (liver, skeletal muscle and adipose tissue)^33^,^34^,^35^,^36^,^37^,^38^. On the other hand, studies on nNOS KO mice have demonstrated its crucial role in regulating metabolism of only peripheral organs excluding liver^33^,^39^. Previous literature and data from hepatic, adipose tissue nitrite and eNOS, nNOS gene expressions associate tissue specific as well as systemic IR in KO mice, while no change in skeletal muscle nitrite and NOS expression correlated with their normal insulin signaling. Reduction in hepatocyte, adipocyte NO synthesis due to attenuated eNOS and nNOS expression in the absence of iNOS, seems to be responsible for decreased NO and systemic IR in iNOS KO mice^3^. On the other hand, our previous experiments demonstrated, the crucial role of hepatic and adipose tissue metabolic deregulation as a predisposing factor for IR in mice lacking iNOS. Kleemann *et al*. demonstrated that HFD induced IR is a tissue dependent process which starts with liver and culminates in white adipose tissue (WAT)^48^. They observed that IR development is paralleled by tissue-specific gene expression changes, metabolic adjustments, changes in lipid composition, and inflammatory responses in liver and WAT but not in skeletal muscle^48^. Further, they demonstrated that alterations in skeletal muscle are largely opposite to those in liver and WAT, which signifies that pathology of IR involve compromised liver and adipose tissue in the initial stages followed by skeletal muscle in the later stages^48^. Hepatic and adipose tissue IR in iNOS KO mice in the present study goes in line with the statement that they are primarily compromised in IR pathology^48^. Altered NO content and tissue metabolism in liver and adipose tissue of insulin resistant iNOS KO mice signify that reduction in their tissue NO pool, affected the normal utilization of glucose and lipids, thereby pre-disposing iNOS KO mice to systemic and tissue IR.

It is well known that NO is important for mitochondrial biogenesis via peroxisome proliferator activated receptor-γ coactivator 1α (PGC-1α)^3^,^49^. PGC-1α acts as a master regulator of mitochondrial oxidative phosphorylation via upregulation of transcription factors PPAR-α and NRFs, and uncoupling proteins (UCPs) respectively^3^. Augmented mitochondrial UCP2 levels in insulin resistant metabolic organs indicate ATP depletion and mitochondrial dysfunction^50^. Almost similar mitochondrial OCR, ATP levels and UCP2 gene expression (Fig. 8f,g) were observed in WT and iNOS KO mice indicating a normal mitochondrial function. This might be probably due to non-stimulation of PGC-1α expression and its downstream PPAR-α is not regulated within 5 weeks of LFD feeding leading to mitochondrial dysfunction^50^,^51^. It could be that PGC-1α up-regulation might not necessarily be dependent only on NO signaling pathways^39^,^52^ or requires more time to affect the downstream pathways leading to change in the mitochondrial function. Mitochondrial OCR levels in KO mice were unaffected after feeding with 5 weeks LFD, thus suggesting mitochondrial dysfunction is not initiated, during the initial stages of IR. We demonstrated that systemic and tissue IR in iNOS KO mice is a resultant of perturbed glucose and lipid availability affecting insulin signaling along with altered tissue NO availability, but not due to mitochondrial dysfunction. Studies involving fat enriched obesogenic diets in WT and iNOS KO mice on mitochondrial functionality and incidence of IR at later time points might further help to understand the role of NO on mitochondrial performance. Therapeutic efficacy of dietary supplements containing NO donors (NaNO_2_) or its substrates (L-Arginine) were extensively studied^53^,^54^,^55^,^56^,^57^,^58^. Administration of NaNO_2_ (50–150 mg/L) in drinking water for 10 days/4 weeks have shown to improve insulin resistance parameters in db/db and KKAy KO mice models of type 2 diabetes^54^,^55^. Similarly, dietary supplementation of L-Arginine to rodents or patients with metabolic syndrome and obesity improved systemic IR and energy homeostasis^53^,^56^,^57^,^58^. Systemic IR due to reduced NO levels in iNOS KO mice suggests usefulness of NO donors/L-arginine to treat IR, obesity and metabolic syndrome. Studies involving the nitrite pharmacotherapy for treating insulin resistant iNOS KO mice are out the scope of the present study which needs future investigations. In summary, the present study thus suggests importance of optimal iNOS along with eNOS and nNOS in the maintenance of glucose/lipid metabolism and insulin sensitivity.

## Materials

RevertAid H Minus first strand kit for cDNA synthesis and (2X) PCR master mix were obtained from Fermentas Life Sciences (Vilnius, Lithuania). Maxima SYBR Green (2X) RT-PCR Master Mix was purchased from Roche Applied Science (Lewes, UK). IRS-1(C-20, sc-559), pIRS-1 (Ser 307) (sc-33956) antibodies were purchased from Santa Cruz Biotechnology (Santa Cruz, USA) while AKT-1/2/3 (11E7, 4685), pAKT-1/2/3 (Ser 473) (D9E, 4060) and GAPDH (14C10, 2118) antibodies were procured from Cell Signaling Technology (Danvers, MA). All the primers used in the study were from Integrated DNA technology (India). Human insulin (Humulin, 5000 IU/ml) was from El Lilly, USA and rest of the chemicals including antibody against β-Actin (AC-15, A3854) were from Sigma Aldrich Co. (St. Louis, USA).

## Methods

### Animals

Eight week old male C57BL/6 WT and iNOS KO mice (Jackson Labs; 002609) were housed in IVC cages (Tecniplast, Italy) at 24 ± 2 °C with free access to food and water. Experiments were approved by the Institutional Animal Ethics Committee, Council for Scientific and Industrial Research-Central Drug Research Institute (CSIR-CDRI) (IAEC/2014/43) and conducted in accordance with the Guidelines of Committee for the Purpose of Control and Supervision of Experiments on Animals (CPCSEA). Mice used in the present study were randomly genotyped as described earlier^59^,^60^. Animals were kept for five weeks on LFD [Research diets, D12450H], which contains 10% kcal fat which is 35% less compared to HFD (4057 kcal% energy). Body weight in each group was measured weekly from day zero to the completion of five weeks.

### Body composition analysis

Mice were analyzed by echo MRI for body composition (E26-226-RM Echo MRI LLC, USA), by applying radio frequency pulses at a distinct static magnetic field as described earlier^61^. Each mouse during the measurement period was placed into a thin wall plastic cylinder (4.5 cm diameter) allowing only limited vertical and horizontal movement.

### Indirect calorimetry

Metabolic measurements were performed in conscious and unrestrained WT, KO mice using six station oxymax CLAMS [CLAMS-CIS-6 with CLAMS-C-6MR, Columbus Instruments, USA] as described earlier^62^. The system was calibrated against a standard gas mixture to measure O_2_ consumed (VO_2_, ml/kg/h) and CO_2_ generated (VCO_2_, ml/kg/h). Metabolic rate (VO_2_) and respiratory exchange ratio (RER) (VCO_2_/VO_2_, mL/kg/min) were evaluated over a 3-day period. Energy expenditure (Kcal/h; heat production) was calculated using a rearrangement of the Weir equation as per the instruction of the manufacturer [(3.815 + 1.232*RER)*VO_2_ (A mean of 50 values per mouse)].

### Serum parameters

Mice were categorized in fasted (overnight, 6 h) and ad libitum fed groups to measure their circulating lipids [total cholesterol (TC), triglycerides (TG), low and high density lipoprotein (LDL, HDL)] using commercial kits from Randox, UK. Insulin levels were measured by using ELISA kits (Crystal Chem, USA); while ALT, total bilirubin and creatinine were monitored using kits from Merck, Germany. Effect of fasting was assessed at two time points (6 h, overnight) as defined earlier^7^.

### Tolerance tests

Mice fasted for 6 h were administered 2 g/kg D-Glucose, 2 g/kg sodium pyruvate and 0.6 IU/kg insulin by the intraperitoneal (i.p.) route to assess glucose (GTT), pyruvate (PTT) and insulin tolerance tests (ITT) respectively, as per the protocols used earlier^63^. Blood glucose was monitored using glucometer (Roche Diagnostics, India) at 0, 15, 30, 60 and 120 min after administration of either glucose, pyruvate or insulin injection. Indices of insulin sensitivity/IR, beta cell (HOMA-IR, HOMA-B) functionality and the quantitative insulin check index of insulin sensitivity (QUICKI) were calculated as per the formulae used by other investigators^21^,^22^.

### Tissue biochemistry

The fasted mice (6 h) were sacrificed after anesthesia with ether to retrieve the organs. Liver triglyceride was measured using Abcam Triglyceride quantification kit (Abcam, ab65336) and cholesterol using Amplex Red Assay kit (Invitrogen, A12216). Tissue glycogen was analysed using Biovision (K 646–100) kit. ATP levels in hepatic and adipose tissue homogenates were estimated calorimetrically using ATP assay kit (Abcam, ab83355) as per manufacturer’s instructions.

### Hematoxylin and Eosin (HE) staining

Formalin fixed tissues were processed in graded concentrations of ethanol followed by xylene prior to liquid paraffin infiltration^64^. The paraffin embedded tissue blocks were sectioned (4–5 μm slices), HE stained images were captured in the bright field mode using an up-right microscope (DM5000, Leica Microsystems, Germany).

### RNA isolation and Real time PCR

Quantitative gene expression analysis was performed using SYBR Green as described previously^65^. Briefly, total RNA was extracted using TRIZOL reagent, and cDNA was synthesized using RevertAid H Minus first strand cDNA synthesis kit. Real-Time PCR (qPCR) for various genes was performed using LightCycler 480II Real-Time PCR system (Roche Applied Science, Indianapolis, IN). 18S rRNA was used as reference gene for normalization to calculate the expression of candidate genes. List of various primers used for qPCR analysis has been shown in Table S1.

### Immunoblotting

Protein samples (40 μg) were run on 8% SDS-PAGE, transferred to polyvinylidene difluoride (PVDF) membranes (Amersham), blocked with 5% BSA in TBST (for 2 h, at RT)and then probed with primary antibodies [diluted in 1:1 ratio of TBST (pH 7.4) and 5% BSA, incubated overnight at 4 °C]. Membranes were later incubated with horse radish peroxidase-linked secondary IgGs (1:10,000) for 2 h at RT, washed and visualized using ECL detection kit (Amersham Biosciences, UK). The signals were captured and normalized with GAPDH for liver and skeletal muscle proteins, or β-actin for adipose tissue proteins, to assess the fold change with respect to WT controls^66^.

### Total nitrite estimation

Plasma and tissue total nitrite (nitrate and nitrite) were estimated using Griess reagent method^67^. Pre-activated cadmium pellets were used for reduction of 100 μl of plasma or 100 mg tissue (liver, adipose and skeletal muscle) homogenates for 4 h at RT. Equal volumes (1:1) ratio of supernatant and Griess reagent were incubated for 30 min (37 °C in dark), followed by deprotinization with 3% trichloroaceticacid (TCA) and reading at 545 nm. Concentration of test samples total nitrite was calculated using sodium nitrite as standard.

### Mitochondrial respiratory measurements

Tissue mitochondria isolation followed by oxygen consumption rates measurements (OCR, a reliable indicator of mitochondrial function) were performed as recommended by Seahorse Biosciences^68^. Briefly, hepatic and adipose tissues were minced in ~10 volumes of mitochondrial isolation buffer (MIB, pH 7.2) followed by homogenization in dounce homogenizer. Tissue homogenates were initially centrifuged at 800 g to remove the cell debris followed by its supernatant at 8000 g for 10 min at 4 °C. The pellets obtained were washed twice in MIB and finally resuspended in 1X mitochondrial assay buffer (MAS, pH 7.2) buffer with 10 mM (pH 7.2) succinate. Equal quantity of mitochondria was normalized with the equal mitochondrial protein content using BCA reagent. After incubation at 37 °C for 8–10 min, plates were placed in XFp Bioanalyzer, and mitochondrial OCR was measured before and after sequential addition of respiratory reagents like 4 mM ADP, 3.16 μM Oligomycin, 4 μM FCCP, and 4 μM Antimycin A^68^.

### Statistical Analysis

Data has been summarized as mean ± SEM (standard error of the mean). Groups were compared by independent Student’s t test or Mann-Whitney U test wherever applicable. Groups were also compared by two way analysis of variance (ANOVA) and the significance of mean difference between the groups (WT and KO) was done by Bonferroni post hoc test after adjusting for multiple contrasts. Prior to analysis, data was ascertained for normality and homogeneity of variance by Shapiro-Wilk’s test/Kolmogorov-Smirnov test and Levene’s test respectively. A two-tailed (*α* = 2) p < 0.05 was considered statistically significant. Analyses were performed on STATISTICA software (Version 7.1 StatSoft, Inc. USA) and graphs were made on GraphPad Prism (Trail version 5.01).

## Conclusion

Previous studies on iNOS KO mice, using HFD with prolonged regimens demonstrated protection against systemic IR. However, the present study even after using LFD only for 5 weeks, observed glucose intolerance, enhanced hepatic glucose production, lipid accumulation, reduced circulatory and tissue nitrite, IR and altered expression of the genes involved in the NO synthesis, lipid and glucose metabolism in iNOS KO mice. Interestingly iNOS mice did not exhibit any change in physical activity but VCO_2_, RER, and heat production were significantly less, further suggesting altered metabolism. We for the first time report systemic IR without alteration in the serum cytokines, ruling out the pivotal role of inflammation in IR. We also did not observe skeletal muscle IR but found hepatic and adipose tissue IR in iNOS KO mice. Taken together, IR in iNOS KO, imply crucial role of NO in the regulation of energy and metabolic homeostasis.

## Additional Information

**How to cite this article:** Kanuri, B. N. *et al*. Altered glucose and lipid homeostasis in liver and adipose tissue pre-dispose inducible NOS knockout mice to insulin resistance. *Sci. Rep.* **7**, 41009; doi: 10.1038/srep41009 (2017).

**Publisher's note:** Springer Nature remains neutral with regard to jurisdictional claims in published maps and institutional affiliations.

## Figures and Tables

**Table 1:** Biochemical parameters in 5 weeks LFD fed WT and KO mice.

S. No.	Parameter	5 weeks					
		6 h fasted		Overnight fasted		Fed (ad libitum)	
		WT	KO	WT	KO	WT	KO
1	TG (mg/dl)	81.55 ± 3.35	178.37 ± 6.96^***^	79.42 ± 7.07	238.29 ± 32.25^***^	104.26 ± 8.10	151.09 ± 9.03^**^
2	TC (mg/dl)	99.22 ± 3.59	175.87 ± 6.38^***^	82.51 ± 6.69	256.09 ± 17.55^***^	133.92 ± 7.67	178.08 ± 15.55^*^
3	LDL (mg/dl)	22.71 ± 1.70	25.36 ± 2.21	44.31 ± 3.25	56.19 ± 7.38	17.54 ± 1.43	18.95 ± 1.11
4	HDL (mg/dl)	34.45 ± 2.93	32.66 ± 2.34	43.72 ± 3.11	51.27 ± 6.43	23.14 ± 1.37	26.94 ± 1.70
5	NEFA (mmol/L)	0.92 ± 0.04	1.69 ± 0.07^***^	1.45 ± 0.14	3.25 ± 0.11^***^	0.74 ± 0.04	1.00 ± 0.09^*^
6	Glucose (mg/dl)	142.13 ± 2.41	166.38 ± 5.61^**^	112.75 ± 5.36	126.63 ± 5.07	182.88 ± 4.82	197.38 ± 7.01
7	Insulin (ng/ml)	0. 69 ± 0.03	1.08 ± 0.13^*^	0.43 ± 0.03	0.54 ± 0.05	1.49 ± 0.06	3.40 ± 0.48^**^
8	HOMA-IR	—	—	2.62 ± 0.22	3.79 ± 0.49*	—	—
9	HOMA-B	—	—	75.96 ± 10.34	68.35 ± 5.53	—	—
10	QUICKI	—	—	0.33 ± 0.0038	0.32 ± 0.0054*	—	—
11	ALT (IU/L)	31.55 ± 2.11	27.20 ± 0.97	31.78 ± 3.19	27.21 ± 1.88	36.53 ± 4.65	27.09 ± 2.29
12	T. Bilirubin (mg/dl)	0.13 ± 0.02	0.11 ± 0.02	0.15 ± 0.01	0.14 ± 0.02	0.16 ± 0.01	0.17 ± 0.01
13	Creatinine (mg/dl)	0.29 ± 0.01	0.24 ± 0.03	0.32 ± 0.02	0.30 ± 0.02	0.28 ± 0.01	0.26 ± 0.01

Results are expressed as mean ± SEM (n = 8–10).
