# Corrigendum: Altered glucose and lipid homeostasis in liver and adipose tissue pre-dispose inducible NOS knockout mice to insulin resistance

**DOI:** 10.1038/srep46010

**Published:** 2017-04-06

**Authors:** Babu Nageswararao Kanuri, Jitendra S. Kanshana, Sanjay C. Rebello, Priya Pathak, Anand P. Gupta, Jiaur R. Gayen, Kumaravelu Jagavelu, Madhu Dikshit

Scientific Reports 7: Article number: 4100910.1038/srep41009; published online: 01 20 2017; updated: 04 06 2017

In the original version of this Article, the link to the Supplementary Information file was omitted. This has now been corrected in the PDF and HTML versions of the paper.

